# CVD Nanocrystalline Diamond Film Doped with Eu

**DOI:** 10.3390/ma15165788

**Published:** 2022-08-22

**Authors:** Elena B. Yudina, Alexander E. Aleksenskii, Sergey A. Bogdanov, Sergey S. Bukalov, Larisa A. Leites, Dmitry B. Radishev, Anatoly L. Vikharev, Alexander Y. Vul’

**Affiliations:** 1 Ioffe Institute, Polytechnicheskaya Str. 26, 194021 St. Petersburg, Russia; 2 Institute of Applied Physics RAS, 46 Ul’yanov Str., 603950 Nizhny Novgorod, Russia; 3 Nesmeyanov Institute of Organoelement Compounds, Vavilova Str. 28, 119991 Moscow, Russia

**Keywords:** CVD diamond film, diamond nanoparticle, Eu ion color centers, rare-earth element photoluminescence

## Abstract

This paper submits experimental results of a study directed towards the formation of Eu ions’ luminescent centers in CVD diamond films. A new approach is based on use of diamond nanoparticles with a surface modified with Eu ions for seeding at CVD growth. Nanocrystalline diamond films (NCD) doped with Eu have been grown from the gas phase on silicon substrates by microwave plasma-assisted CVD at a frequency of 2.45 GHz. The photoluminescence spectra clearly show several electronic transitions of the Eu^3+^ ions, which confirm the incorporation of Eu ions into the NCD film.

## 1. Introduction

Diamond has extremely advantageous properties for high-power, high-frequency electronics, radiation detectors, and field electron emitters among numerous semiconductor materials. Some of diamond’s outstanding electronic properties include: a breakdown field of 10 MV cm^−1^, high saturated drift velocities of 2.3 × 10^7^ cm s^−1^ for electrons and the highest thermal conductivity of any materials, which is important for many power electronics and optoelectronics applications when operating is limited by heat removal [1,2]. 

At present, the microwave plasma chemical vapor deposition (MPCVD) method is typically used for growing various nano- and polycrystalline diamond films, as well as single-crystal diamond layers. The CVD technology has opened the door for diamond doping during its growth, for the creation of NV, SiV, and GeV color centers in CVD diamond for applications in the field of quantum communication and quantum information, as well as for the creation of ultrasensitive sensors of magnetic and electric fields [3,4]. 

Over the last several years, the color centers formed by rare metal impurities in diamonds, firstly Eu [5,6,7,8], have received attention, along with the NV, SiV, and GeV color centers. The reasons for this are their high quantum efficiency and long fluorescence relaxation time: microseconds compared with nanoseconds for NV, SiV, and GeV centers. Generally, only two techniques of NCD film doping with rare metals are used: introducing a stable [6,8] as well as unstable [5] rare metal substance into the film at the seeding stage and ion implantation [9].

The color centers can be used as biomarkers prepared by the incorporation of rare-earth metal ions into CVD diamond layers. However, to create such biomarkers, the problem of CVD diamond doping with impurities of rare-earth elements has not yet been solved. The first step in solving this problem can be the production of CVD nanocrystals of diamond doped with rare metal ions. 

Diamond particles produced by detonation of strong explosives (detonation nanodiamonds, DND) [10,11] opened a new way for the development of CVD technology for diamond films. The concentration of crystallization centers up to 10^12^ cm^−2^ and the growth of diamond films on substrates that have no chemical affinity for carbon has been successfully realized when DND particles sized 4–5 nm were used for seeding [12].

A new possibility of using DNDs in CVD diamond film technology has arisen after experimental evidence of the effects of chemical modification of the surface of DND particles by metal ions [13]. Indeed, such DND nanoparticles grafted with metal ions can be also used for seeding as mentioned above, and it could be expected that metal ions would be introduced into the diamond film during the CVD growth process.

Previous works [5,6,8] have demonstrated the photoluminescence of NCD films grown from seeds containing luminescent europium precursors. In this work, an alternative method is proposed: Eu^3+^ ions coordinated to nanodiamond particles are used as precursors. The present study verifies the abovementioned hypothesis on the incorporation of Eu^3+^ ions into NCD film.

Thus, DND nanoparticles grafted with Eu^3+^ ions have been used as seeding centers for the CVD growth of diamond films. The choice of Eu ions is determined by practical interest in obtaining optoelectronics devices, X-ray scintillators and biomarkers due to the bright luminescence of intracenter optical transitions in such ions [14]. As we mentioned above, only a few successful experiments on the creation of luminescent Eu centers in diamond have been reported [5,6,8]; here, we discuss a new approach for doping CVD diamond films with rare metal ions.

## 2. Materials and Methods

**Figure 1:**
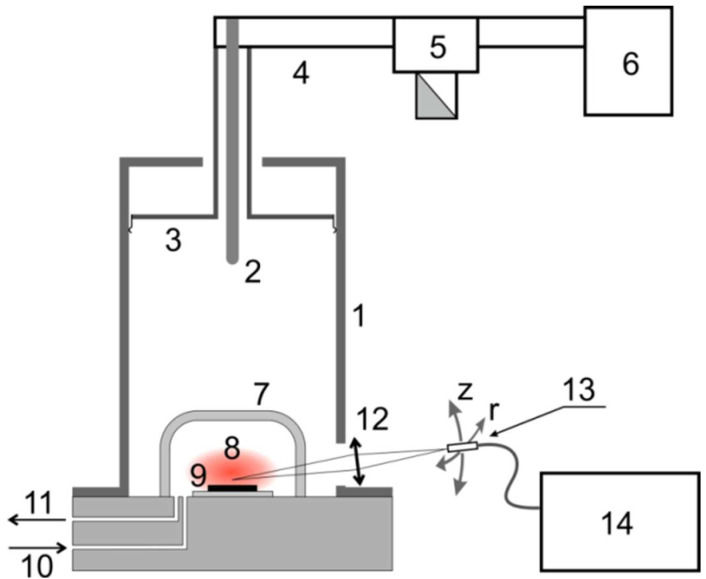
Block-scheme of the CVD reactor: 1—a wall of the microwave resonator; 2—adjustable communication device based on a coaxial waveguide; 3—the upper movable wall of the resonator; 4—rectangular waveguide; 5—circulator; 6—magnetron; 7—quartz flask; 8—plasma; 9—silicon substrate; 10—supply of the gas mixture; 11—pumping out; 12—focusing lens; 13—movable light guide; 14—HORIBA Jobin Yvon FHR-1000 spectrometer.

Nanocrystalline diamond films doped with Eu have been grown from the gas phase on silicon substrates by microwave plasma-assisted chemical vapor deposition at a frequency of 2.45 GHz using a setup similar to that described in [15] (see Figure 1).

A plasma-chemical reactor consists of a cylindrical resonator (1) of a small diameter, in which the TM013 mode is excited by a coaxial waveguide (2). Resonant conditions in the resonator are achieved by moving its upper movable wall (3). The coaxial waveguide is connected to a rectangular waveguide (4) and then through a circulator (5), which is connected to a magnetron (6). A quartz bulb (7) limits the region of existence of a continuous discharge (8).

The following parameters were used at CVD diamond film growing conditions: gas mixture pressure 34 Torr, gas flow 200 cm^3^/min, MW power 1.05 kW, substrate temperature 720 °C, size of monocrystalline silicon substrate with (100) orientation is 20 × 20 mm^2^. The ratio of methane to hydrogen in the gas mixture was CH_4_/H_2_ = 2%. 

It is known that the content of non-diamond carbon at the intercrystalline grains in the diamond films can be reduced by adding oxygen to the hydrogen–methane gas mixture. Oxygen reduces the content of the phase of sp^2^ hybridized carbon atoms and weakens the renucleation processes, improving the quality of diamond films [16]. Therefore, our experiments have been carried out in a gas mixture of H_2_/CH_4_/O_2_ with an O_2_/CH_4_ ratio of 0.25. Regarding safety concerns, the use of an oxygen–hydrogen mixture is not dangerous due to too little oxygen (O_2_/H_2_ = 0.5%). Such mixtures are often used in CVD technology (for the synthesis and etching of diamonds), including those with higher oxygen content. For example, for substrate preparation before growth, some groups use etching in a hydrogen/oxygen mixture, O_2_/H_2_ = 2% [17].

For the synthesis of not doping CVD diamond films, a DND hydrosol similar to the previous one [18] has been used. Industrial DND powder produced by FGUP SKTB “Technolog” (St. Petersburg) has been additionally purified from residual impurities, annealed in an air atmosphere at 450 °C, subjected to ultrasonic treatment, and then centrifuged, resulting in a stable hydrosol containing free 4–5 nm diamond particles being obtained.

DND particles with a surface modified with Eu ions (DND-COO-{Eu}) [13] were used for seeding upon growing a Eu-containing diamond layer. Grafting of Eu^3+^ ions to the DND surface was performed by mixing an aqueous DND suspension with an aqueous solution of Eu nitrate with constant stirring. The resulting reaction mixture was decanted by centrifugation. The Eu content in the decantate was then detected by the Arsenazo indicator. According to the results of elemental analysis, the Eu content in DND-COO-{Eu} samples was ca. 1.3 wt%.

**Figure 2:**
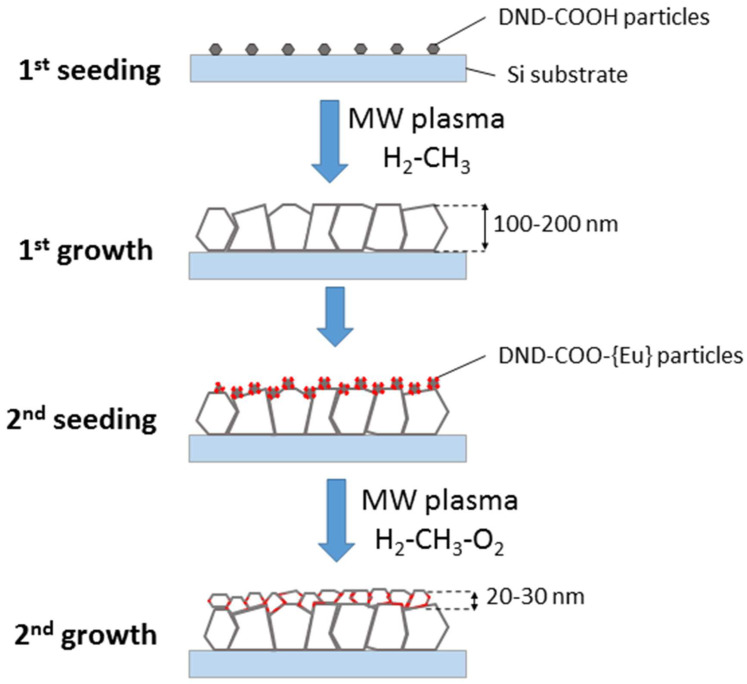
Scheme for synthesis of CVD diamond film doped with Eu.

The growth of CVD diamond films doped with Eu was carried out in four stages (Figure 2):

1. The first stage was the seeding of a silicon substrate with diamond powder using a DND suspension (conc. 0.69 wt.%). The DND hydrosol was sonicated for two hours. All silicon substrates were preliminarily cleaned in acetone in an ultrasonic bath, then ultrasonicated in hydrosol for two hours.
2. The products from the first stage of growing a silicon substrate were placed in a CVD reactor. DND particles acted as nucleation centers during the growth of a nanocrystalline diamond film. The duration of the diamond film growth process was one hour; as a result of the growth, the film thickness was 300 nm. The film thickness was measured by the method described in the previous paper [12].
3. In the second stage, the surface of nanocrystalline diamond film was treated with nanodiamond particles with a surface modified with Eu ions. The concentration of the DND-Eu particles in hydrosol was 1.43 wt.%.
4. Then, in the second stage, growth of a diamond film on DND-Eu particles was completed. The overgrowth time was 10 min and the thickness of diamond film doped with Eu did not exceed 50 nm.

SEM images of the CVD film surface were obtained using Tescan SBH3. The accelerating voltage of the electron beam was 30 kV. The pressure in the measuring chamber was about 10^−7^ Torr.

Photoluminescence and Raman analysis were carried out using a LABRAM-300 spectrometer (Horiba-Jobin-Yvon, France). Photoluminescent spectra at various points of the CVD film were studied with an excitation wavelength of 532 nm and 638.8 nm. The Raman spectra were obtained in the range 100–3500 cm^−1^ with a resolution of 1 cm^−1^ at laser excitation with wavelengths 632.8 nm of ~1 mW. The power was deliberately reduced to avoid sample heating under the laser beam.

## 3. Results and Discussion

**Figure 3:**
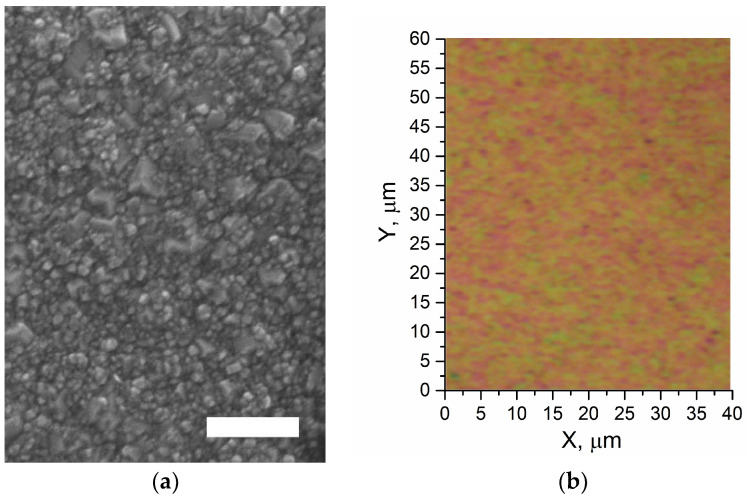
SEM, scale bar is 0.5 μm (**a**), and optical image of the CVD DND(Eu) film surface (**b**). The image (**b**) has been done at magnifying 50.

The SEM image of the CVD film grown with DND (Eu) is provided in Figure 3. The duration of film growth in the first layer was one hour. The image shows both large crystals with a size of 200–300 nm and small crystals of about 20 nm. Large crystals clearly belong to the first layer according to the scheme in Figure 2. Smaller crystals (20–30 nm) are the result of the growth of DND-Eu particles deposited on the first layer. Thus, the second layer does not form a continuous film but represents separate and intergrown crystallites, partially or completely covering the submicron diamonds of the first layer.

The surfaces of the grown CVD films have different colors at different points of the surface (Figure 3b), which is probably a result of variant impurity levels in different film spots. The latter suggestion is followed from differences in photoluminescence spectra of the surface regions of different colors. 

**Figure 4:**
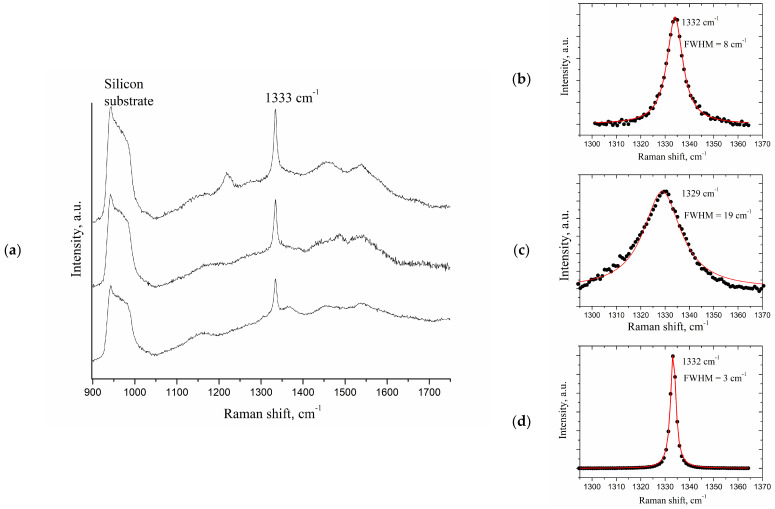
Raman spectra of various points of the CVD DND (Eu) film in different regions of CVD film (**a**). It is shown for comparison the diamond lines (1332 cm^−1^) for CVD DND (Eu) film (**b**), DND powder (**c**) and microsized diamond particles (**d**) measured using the same experimental set-up on a very large scale.

Raman spectra of different points of the CVD DND (Eu) film is presented in Figure 4. Variations in spectra from point to point indicate inhomogeneity of the NCD film. The origin of the Raman peak around 1220 cm^−1^ is unclear and is proposed to be side-products of CVD process. All of the spectra demonstrate the typical diamond maximum at 1332 cm^−1^, which is characteristic of the T_2g_ vibrational mode of the diamond lattice. Similar to the other polycrystalline CVD diamond films and diamond nanocrystals, this band shows a significant difference in half-widths with the Raman maximum of a microsized single crystal cubic diamond (ca. 3 cm^−1^).

Broadening and shifting of the diamond line into higher frequencies observed for DND powder are well-known and associated with the phonon confinement model in nanoparticles [19]. In contrast to nanoparticles, CVD diamond films do not possess such features, and thus, only a small line broadening indicates some stress in the film. This line broadening is caused by random stress as thoroughly studied and typical for diamond films grown on substrates with lattice parameters different from that of diamond [20]. Besides the specific diamond line (1332 cm^−1^) and the band of the silicon substrate (940–1000 cm^−1^), broad bands are observed in the film, which change from point to point in the region of 900–1750 cm^−1^.

Turning to the results of the study of photoluminescence, we note, firstly, that strong photoluminescence has been observed in the CVD films obtained after seeding with nanodiamond particles grafted with europium ions. 

**Figure 5:**
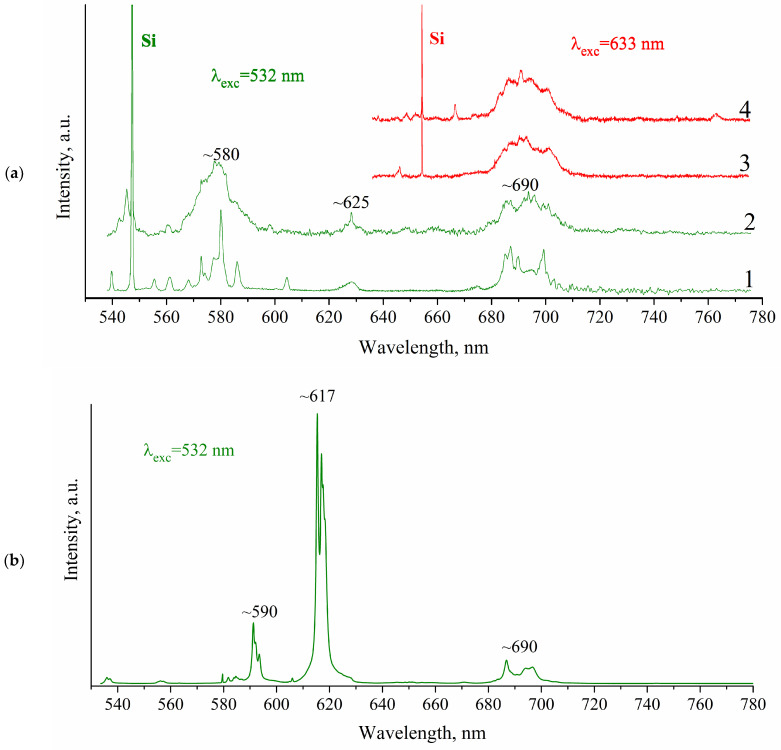
(**a**) Photoluminescence spectra CVD diamond film grown with suspension DND-COO-{Eu} at different excited laser wavelengths: λ_exc_ = 532 nm and λ_exc_ = 633 nm. 1–4—different points on of CVD DND(Eu) surface. (**b**) Photoluminescence spectrum of the europium nitrate powder Eu(NO_3_)_3_ 6H_2_O used in preparation of DND-COO-{Eu} particles suspension. The background has been subtracted in both cases. Band ~ 580 nm corresponds to ^5^D_0_—^7^F_1_ transition, ~625 nm—^5^D_0_—^7^F_2_ transition and ~690 nm—^5^D_0_—^7^F_4_ transition.

As can been clearly seen in Figure 5, Raman spectra of CVD diamond films, grown using Eu-grafted DND suspensions, demonstrate photoluminescence originating by intraconfigurational electronic transition of Eu^3+^ ions.

It is noteworthy that excitation at 633 nm leads to Eu^3+^ emissions in the region of 680–710 nm regardless of any area being excited and its color. This photoluminescence band is assigned to ^5^D_0_→^7^F_4_ of Eu^3+^ as stated, for instance, by V. Sedov et al. [6].

Thus, one can explicitly correspond detected photoluminescence to electronic transitions of Eu^3+^ ion. Furthermore, spots differing in colors throughout the diamond polycrystalline film (Figure 3b) indicate that Eu^3+^ doping is more likely to occur at grain boundaries, where sp^3^ hybridized orbitals of C atoms are transformed to less “tough” sp^2^ one, as proposed by D.E.P. Vanpoucke et al. [14].

Future developments in the use of Eu-grafted DND particles in doping CVD diamond film are aimed at enhancing photoluminescence signal.

## 4. Conclusions

In our research, DND particles grafted with Eu^3+^ ions (DND-COO-{Eu}) were used for the synthesis of a Eu-containing nanocrystalline diamond layer. DND particles with europium were attached to the 300 nm-thick CVD-grown nanocrystalline diamond film by ultrasonic treatment. The subsequent NCD film overgrowth resulted in the formation of a Eu-containing diamond layer. Europium ions were not etched away from the surface and remained present in the film. The results of the photoluminescence analysis clearly showed several electronic transitions of the Eu^3+^ ions, which confirmed the incorporation of europium into the NCD film. Thus, the successful creation of a europium luminescence centers in nanocrystalline diamond film was achieved using the CVD growth and DND particles grafted with Eu^3+^ ions.

## Data Availability

Not applicable.
